# Sclerotherapy embolism: a novel etiology for chronic thromboembolic pulmonary disease

**DOI:** 10.1186/s12890-025-04052-7

**Published:** 2025-12-10

**Authors:** Çağatay Çetinkaya, Altuğ Sağır, Ayşen Terzi, Nezih Onur Ermerak, Şehnaz Olgun Yıldızeli, Bülent Mutlu, Bedrettin Yıldızeli

**Affiliations:** 1 Department of Thoracic Surgery, Uskudar University School of Medicine, Istanbul, Turkey; 2 Department of Cardiovascular Surgery, Uskudar University School of Medicine, Istanbul, Turkey; 3 Department of Pathology, Memorial Sisli Hospital, Istanbul, Turkey; 4 Department of Thoracic Surgery, Marmara University School of Medicine, Istanbul, Turkey; 5 Department of Pulmonology, Marmara University School of Medicine, Istanbul, Turkey; 6 Department of Cardiology, Marmara University School of Medicine, Istanbul, Turkey; 7 Department of Thoracic Surgery, Memorial Atasehir Hospital, Istanbul, Turkey

**Keywords:** CTEPH, Sclerotherapy, Pulmonary endarterectomy, Non-thrombotic obstruction

## Abstract

**Background:**

Chronic thromboembolic pulmonary disease (CTEPD) includes both chronic thromboembolic pulmonary hypertension (CTEPH) and disease without pulmonary hypertension. Although the main cause of CTEPH is mostly due to thromboembolic events, other rare non-thrombotic etiologies may also contribute to chronic pulmonary artery obstruction. This case series presents a unique observation of foreign material embolization related to prior sclerotherapy procedures, confirmed histopathologically after pulmonary endarterectomy (PEA).

**Methods:**

From a prospectively maintained database of 1,105 patients undergoing PEA between 2011 and 2025, four patients (three women and one man; median age 32.5 years, range: 28–41) with a history of sclerotherapy were identified. All underwent sclerotherapy for varicose vein treatment. They were referred for surgery with a preoperative diagnosis of CTEPD, with or without pulmonary hypertension. The final diagnosis was confirmed by histopathological examination of surgical specimens.

**Results:**

All four patients had segmental or lobar perfusion defects and vascular obstruction consistent with organized embolic material. Preoperative mean pulmonary artery pressure (mPAP) was 24.3 ± 7.4 mmHg, and mean pulmonary vascular resistance (mPVR) was 219.3 ± 104.6 dyn·s/cm⁻⁵. Although the surgery was challenging because of difficulty establishing dissection plane, no perioperative morbidity or mortality occurred. Postoperative hemodynamic improvement was observed, with mPAP reduced to 16.3 ± 1.5 and mean PVR to 119.3 ± 45.8 dyn·s/cm⁻⁵ (*p* > 0.05). The mean six-minute walk test distance increased from 381.5 ± 63.2 m preoperatively to 470.0 ± 66.8 m after surgery (*p* > 0.05). Histopathological analysis confirmed the presence of sclerotherapy-related foreign material in all cases. All patients had unilateral lobar obstruction. During a median follow-up of 50 months, no mortality or recurrence of symptoms or pulmonary hypertension was observed.

**Conclusions:**

This report is the first case series to document a direct histopathological link between sclerotherapy and chronic pulmonary artery obstruction. These findings emphasize the need to consider iatrogenic etiologies in patients with unexplained pulmonary vascular disease and support the diagnostic and therapeutic value of PEA in selected cases.

**Supplementary Information:**

The online version contains supplementary material available at 10.1186/s12890-025-04052-7.

## Background

Chronic thromboembolic pulmonary hypertension (CTEPH) is a form of pulmonary hypertension (PH) characterized by persistent obstruction of the pulmonary arteries due to organized thromboembolic material [1]. It is a potentially life-threatening condition for which pulmonary endarterectomy (PEA) remains the only curative option in appropriately selected surgical candidates [2]. This surgical intervention can significantly improve hemodynamics, exercise capacity, and survival in appropriately selected patients.

Although CTEPH is most commonly attributed to unresolved thromboembolic events following acute pulmonary embolism, not all cases are thromboembolic in origin. According to the latest ERS/ESC PH guidelines [3], CTEPH is named as Group IV PH and it has been divided as CTEPH due to the pulmonary embolism and other causes.

Several conditions can mimic CTEPH, including pulmonary artery sarcoma, hydatid cysts, vasculitis, sarcoidosis, fibrosing mediastinitis, pulmonary vein stenosis or occlusion, in situ thrombosis, malignancies, and congenital anomalies of the pulmonary arteries. However, pulmonary embolism caused by foreign materials is not yet recognized within this spectrum. Sclerotherapy, a widely used treatment for varicose veins, involves the injection of sclerosant agents such as polidocanol or sodium tetradecyl sulfate to induce endothelial damage and vessel obliteration. Although generally considered safe, inadvertent passage of these agents into the systemic or pulmonary circulation may lead to mechanical obstruction and subsequent vascular complications [4].

The aim of this study was to review our experience in the surgical treatment of CTEPH in patients with history of use of sclerotherapy. To the best of our knowledge, this is the first study to document histopathologically confirmed sclerotherapy-related material as a cause of chronic pulmonary artery obstruction. In this article, we report 4 patients who underwent PEA and were subsequently diagnosed with sclerotherapy-related pulmonary artery embolism.

## Methods

### Patient selection and data collection

Between March 2011 and May 2025, PEA was performed in 1,105 patients at our center. Among these, four patients (three women and one man) were surgically treated for CTEPD (with or without PH), and were subsequently found to have sclerotherapy-related foreign material in the pulmonary vasculature upon histopathological examination.

All surgeries were performed by the same experienced surgical team. All patients were evaluated and managed within a dedicated multidisciplinary team specializing in CTEPH and PEA, including pulmonologists, cardiologists, radiologists, anesthesiologists, and an expert PEA surgeon.

Demographic characteristics, comorbidities, clinical symptoms, prior history of sclerotherapy, preoperative pulmonary function test results, radiologic and hemodynamic findings, intraoperative and postoperative complications, length of ICU and hospital stay, 30-day mortality, and long-term follow-up data were collected prospectively and analyzed retrospectively. Operative mortality was defined as death occurring during hospitalization or within 30 days postoperatively.

The diagnosis of CTEPD (with or without pulmonary hypertension) was established based on persistent symptoms despite at least three months of anticoagulation therapy. In all patients, chronic vascular obstruction was confirmed by imaging studies, including pulmonary computed tomography (CT) angiography and ventilation-perfusion (V/Q) scans showing segmental perfusion defects. The patient without pulmonary hypertension (CTEPD) underwent surgery due to significant anatomical obstruction and ongoing symptoms despite optimal medical therapy. All patients underwent comprehensive preoperative evaluation, including pulmonary function testing, CT angiography, right heart catheterization, and six-minute walk testing.

Known risk factors for the development of CTEPH include deficiencies in protein C, protein S, and antithrombin III, as well as conditions such as antiphospholipid syndrome, systemic lupus erythematosus, inflammatory bowel disease, splenectomy, and malignancies [5, 6]. In our series, all patients were evaluated for these predisposing conditions, and none exhibited any identifiable thrombophilic or autoimmune disorders.

### Surgical procedure

Our operative protocol has been previously reported [2]. Briefly, surgery was performed under general anesthesia, cardiopulmonary bypass, and deep hypothermia via median sternotomy. In all four patients, the procedure was performed unilaterally, with endarterectomy limited to the affected pulmonary artery. Organized embolic material was meticulously removed, extending into segmental and subsegmental branches.

### Postoperative assessment and follow-up

Early postoperative assessment included right-sided heart catheterization after PEA. Follow-up evaluations were performed at 1, 3, and 6 months postoperatively, including transthoracic echocardiography and six-minute walk tests.

Ethical approval for this study was obtained from the institutional review board, and the study was conducted in accordance with the Declaration of Helsinki.

### Histopathological examination

All excised specimens were submitted for histopathological and immunohistochemical evaluation. PEA specimens were fixed in 10% neutral buffered formalin, processed routinely, and embedded in paraffin. Sections 3–5 μm thick were cut from the blocks and stained with hematoxylin and eosin for routine histological evaluation under a light microscope.

To identify macrophage infiltration and granulomatous reactions, CD68 immunohistochemical staining was performed. CD68 staining was carried out using a monoclonal antibody (DAKO, Agilent Technologies, Glostrup, Denmark). Antigen retrieval was achieved via microwave treatment in citrate buffer (pH 6.0). The staining procedure was conducted using the DAKO EnVision detection system, and visualization was achieved with diaminobenzidine chromogen. Counterstaining was performed with hematoxylin. Human lymph node tissue served as a positive control, and negative controls were prepared by omitting the primary antibody.

### Statistical analysis

Statistical analysis was performed using the Wilcoxon signed-rank test due to the small sample size and non-parametric data distribution. A *p* value of < 0.05 was considered statistically significant.

## Results

### Patient characteristics and preoperative assessment

The patient cohort consisted of three females and one male, with a median age of 32.5 years (range: 28–41 years). Two patients had a history of deep vein thrombosis (DVT) (Table 1). Dyspnea was the predominant symptom in all patients, followed by chest discomfort and fatigue, which were also commonly reported. All patients had a history of sclerotherapy for varicose veins, which was the only common predisposing factor.

**Table 1:** Demographic and clinical characteristics of patients

Age, y (median)	32
Male sex	1 (25%)
Female sex	3 (75%)
Body surface area, kg/m²	26.92
History of DVT	2 (50%)
Smoking history	0
Preoperative mPAP (mmHg)	27.2

**Fig. 1:**
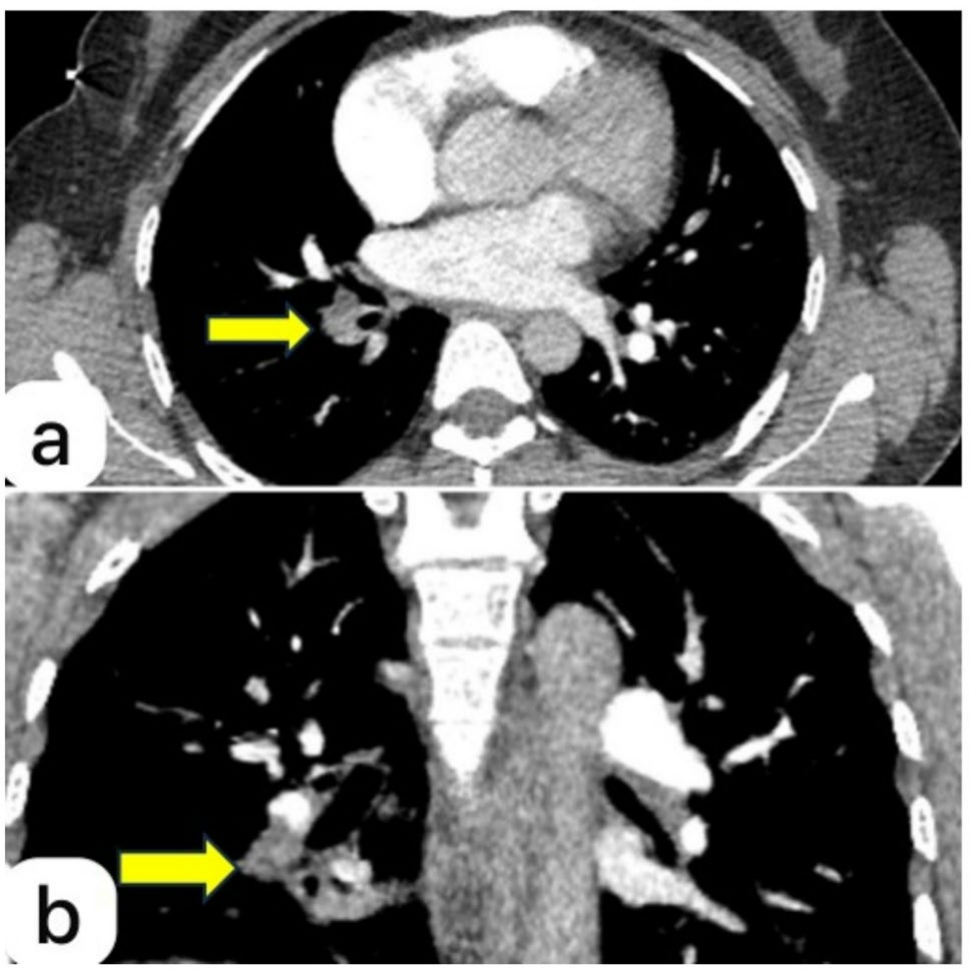
Axial (**a**) and coronal (**b**) computed tomography angiography images of the fourth patient showing an obstructive lesion (yellow arrows) in the segmental branch of the right pulmonary artery, consistent with chronic embolic material

Perfusion defects were identified on ventilation-perfusion (V/Q) scan in all cases and preoperative CT angiography demonstrated a lobar or segmental pulmonary artery obstruction consistent with organized embolic material (Fig. 1).

Body mass index values ranged from 17.2 to 39.4 kg/m², with a mean of 26.9 kg/m², indicating an overall overweight profile among the patients. Preoperative pulmonary function tests were performed in all patients. The FEV₁ values were 2.8 L (80%), 2.25 L (71%), 3.48 L (86%), and 2.22 L (90%), respectively, indicating preserved or mildly impaired pulmonary function.

Preoperative hemodynamic assessments revealed a mPAP of 24.3 ± 7.4 mmHg (range: 16–31 mmHg). PVR was measured with a mean value of 219.3 ± 104.6 dyn·s/cm⁻⁵ (range: 114–350 dyn·s/cm⁻⁵). All patients underwent standard right heart catheterization for accurate preoperative evaluation (Table 2).

**Table 2:** Comparison of baseline and midterm outcomes after PEA

Characteristics	Pre-PEA (Mean ± SD)	Post-PEA (Mean ± SD)	*p*-value
WHO Class I	0	4	
WHO Class II	1	0	
WHO Class III	3	0	
Six minute walk test (mt)	381.5 ± 63.2	470.0 ± 66.8	0.125
Mean pulmonary arterial pressure, mmHg	24.3 ± 7.4	16.3 ± 1.5	0.125
Mean Pulmonary vascular resistance, dyn/s/cm⁻⁵	219.3 ± 104.6	119.3 ± 45.8	0.125

### Operative findings

**Fig. 2:**
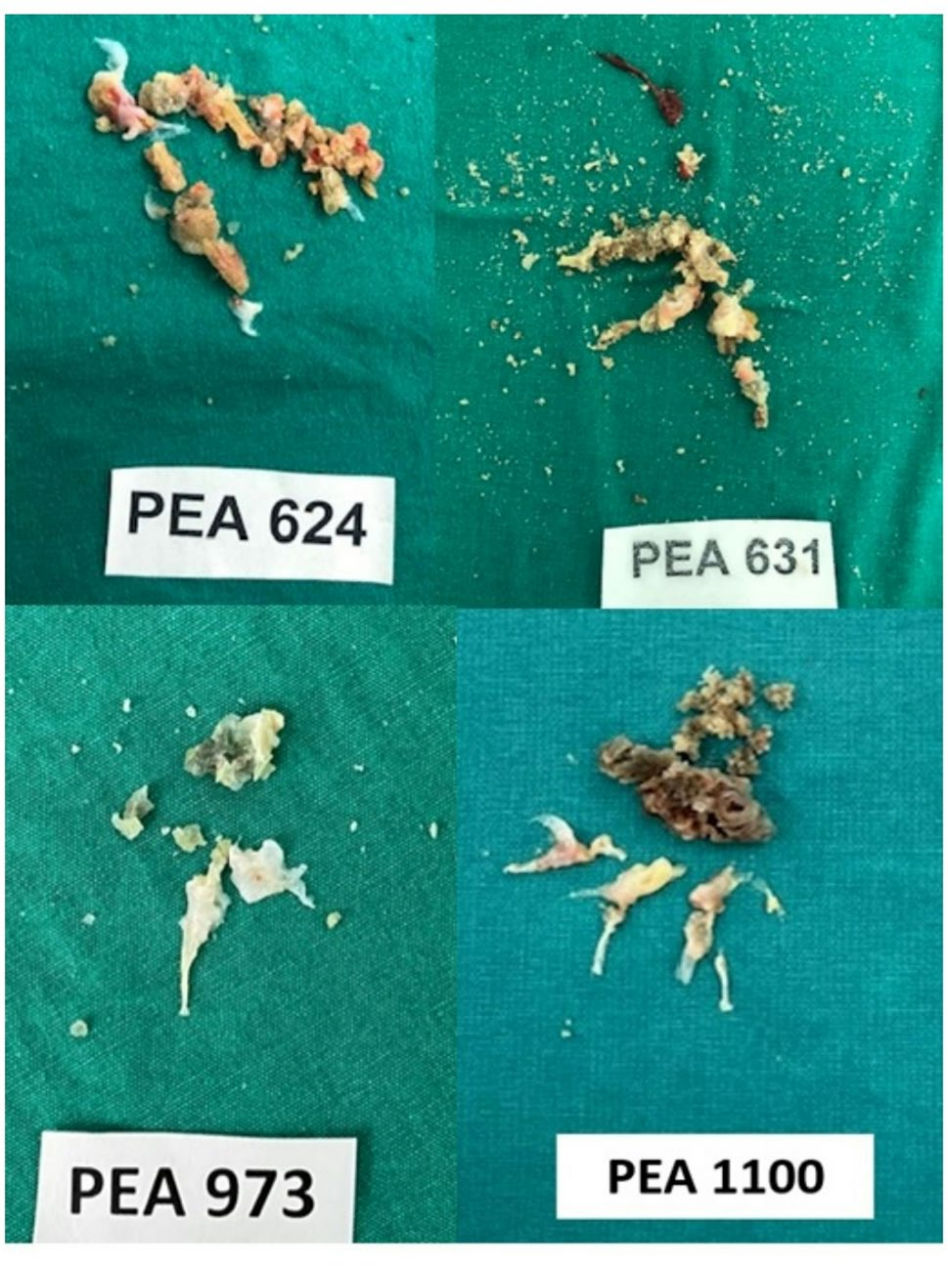
Macroscopic appearance of organized embolic material excised during PEA in all four patients

During surgery, highly calcified organized embolic material, polypoid in nature, was found at the orifice of the lobar pulmonary artery. The specimen was meticulously removed, extending into distal segmental and subsegmental branches, but fragmentation of the specimen also occurred. At the end of the surgery, all segmental and subsegmental branches were patent. Representative images of the excised material from all four patients are shown in Fig. 2, demonstrating the polypoid, fibrotic organized embolic material that caused the chronic intraluminal obstruction.

### Histopathological findings

**Fig. 3:**
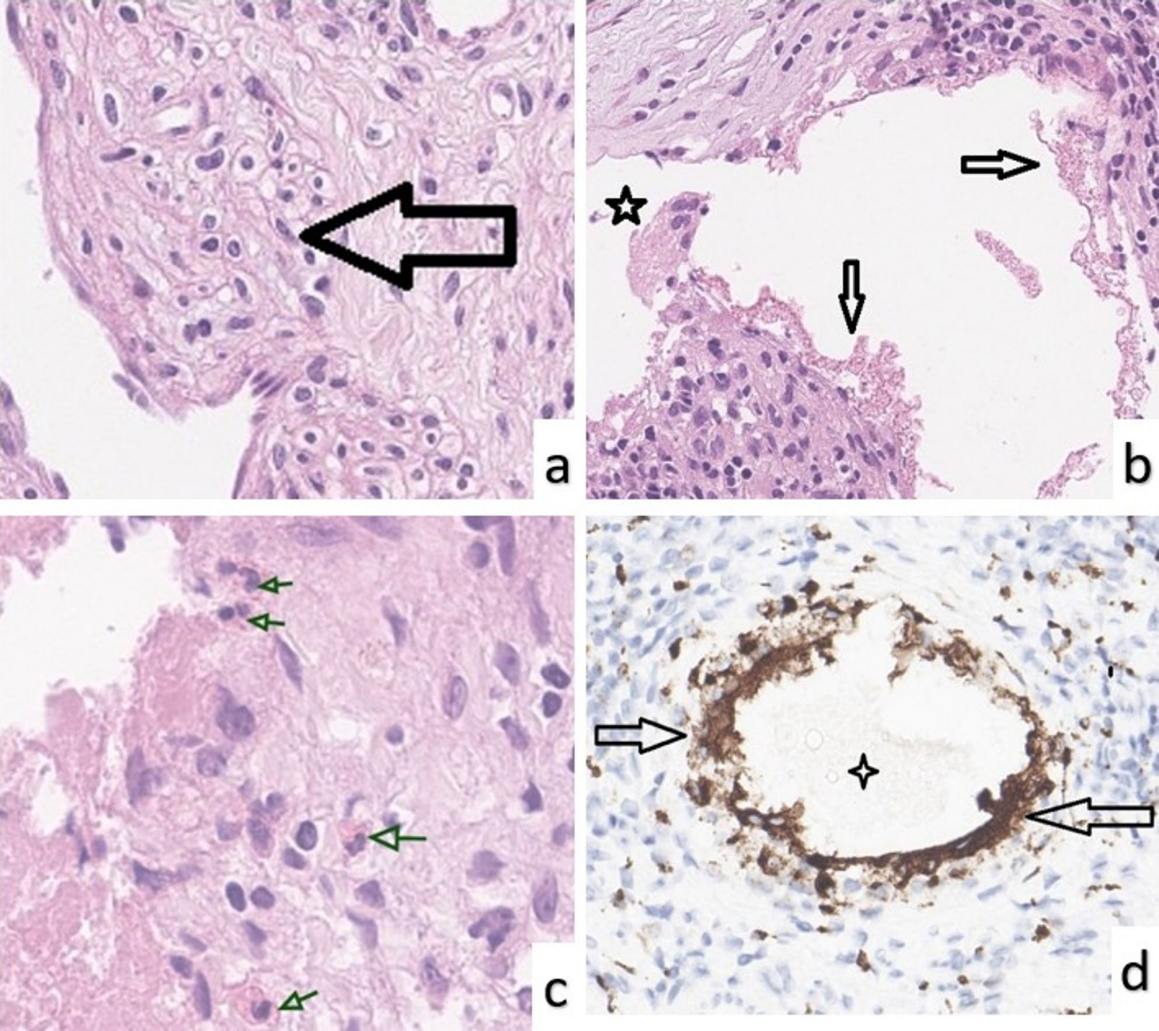
Histopathological findings from PEA specimens. **a** Organized, hyalinized inflammatory thrombus with recanalized vascular-like spaces lined by smooth muscle cells (black arrow). **b** Eosinophilic, granular amorphous material resembling sclerosing agent deposits (black arrows) accompanied by a foreign body-type multinucleated giant cell (asterisk). **c** Granulomatous inflammation with epithelioid histiocytes surrounding amorphous deposits (green arrows), along with eosinophilic infiltration. **d** Granulomatous reaction around amorphous material remnant (asterisk), with surrounding macrophages and multinucleated giant cells showing strong CD68 positivity (black arrows). (Hematoxylin & eosin for 3a–3c; immunohistochemistry for CD68 in 3d; original magnifications ×400–500)

Histological examination of the endarterectomy material revealed extensively organized and hyalinized thromboembolic material within the pulmonary artery. The thrombotic material showed marked fibrous organization and recanalized vascular-like spaces separated by fibrous septa, creating a characteristic “colander-like” appearance [7]. These channels were lined by flattened, endothelial-like cells (Fig. 3a).

In several regions of the thromboembolic tissue, multinucleated giant cells, granulomatous inflammation rich in histiocytes, and eosinophilic, granular amorphous remnants resembling sclerosing agent deposits were observed (Fig. 3b and c). The presence of foreign body-type giant cells in these areas suggested embolization secondary to prior sclerotherapy.

CD68 immunohistochemistry showed strong positivity in histiocytes and multinucleated giant cells within the granulomatous areas, confirming a prominent foreign body-type inflammatory response (Fig. 3d). Representative preoperative CT angiography, excised PEA specimens, and corresponding histopathological images for all four patients are provided in Supplementary Figure S1.

Additionally, moderate chronic inflammatory infiltrates consisting of lymphocytes and plasma cells were observed in perivascular regions and adjacent to the thromboembolic foci. Scattered eosinophilic leukocytes were also noted, becoming more prominent around the amorphous foreign material (Fig. 3c). No neutrophilic infiltration was identified.

### Postoperative outcomes

Postoperative recovery was uneventful. All patients were extubated on the first postoperative day. Two patients required intensive care for two days, while the other two were discharged from the ICU after one day. The length of hospital stay was recorded as 9, 7, 8, and 7 days, respectively, with a mean duration of 7.7 ± 1.0 days (Table 3).

**Table 3:** Specific patient information

Patient No.	Age (y)	Sex	PEA Performed	Hospital stay (days)	History of DVT	Follow up (mo)	Current Status of WHO Class
1	28	F	Unilateral	9	No	71	Class II
2	33	F	Unilateral	8	No	69	Class III
3	32	M	Unilateral	8	Yes	26	Class III
4	41	F	Unilateral	7	Yes	5	Class III

No mortality or morbidity was observed. The patients were followed for a median duration of 50 months (range: 5–71 months). Symptomatically, dyspnea, chest discomfort, and fatigue improved in all patients; at last follow-up all were in WHO functional class I.

Postoperatively, mPAP dropped from 24.3 ± 7.4 mmHg to 16.3 ± 1.5 mmHg. Similarly, PVR improved from 219.3 ± 104.6 dyn·s/cm⁻⁵ to 119.3 ± 45.8 dyn·s/cm⁻⁵ (*p* = 0.125). The mean 6-minute walk distance also increased from 381.5 ± 63.2 m preoperatively to 470.0 ± 66.8 m after surgery, indicating a marked improvement in functional capacity (Table 2). Detailed individual data for all patients, including WHO functional class, hemodynamic parameters, and 6-minute walk distance before and after PEA, are provided in Supplementary Table S1.

## Discussion

Group IV PH is subclassified as obstructions due to thromboemboli or other pulmonary artery obstructions according to recent ESC/ERS guideline [3]. These non-thrombotic obstructions include hydatid cysts [8], vasculitides [9], Behçet’s disease [10], and pulmonary artery sarcoma [11], and other rare intraluminal or extrinsic entities. Our series suggests that sclerotherapy-related foreign material embolization represents a plausible iatrogenic mechanism of chronic intraluminal obstruction within Group IV disease, reinforcing the need to systematically inquire about prior venous interventions when evaluating patients with CTEPD/CTEPH.

Although no prior reports have directly linked sclerotherapy to the development of CTEPD, several publications in the literature highlight the potential for thromboembolic complications following sclerotherapy procedures. A fatal pulmonary embolism has been reported following ultrasound-guided foam sclerotherapy (UGFS) and extensive phlebectomy for varicose veins [12]. The author emphasized that while UGFS is generally considered safe, embolic complications may occur due to systemic migration of the sclerosant foam, and recommended precautions to minimize such risks. These findings, along with our own cases where foreign material consistent with sclerotherapy agents was found within the pulmonary vasculature, suggest that in certain individuals, embolic complications may persist and potentially evolve into chronic vascular obstruction mimicking CTEPH.

A similar case of acute massive pulmonary embolism was reported following high ligation and compression sclerotherapy for varicose veins, further supporting the potential of sclerotherapy procedures to cause serious thromboembolic events [13].

In a large retrospective series involving 2,616 patients undergoing ultrasound-guided foam sclerotherapy, the incidence of pulmonary embolism was reported as 0.3%, suggesting that while acute events are rare, our series of four cases with histologically confirmed chronic embolic obstruction underscores the potential for underrecognized long-term complications [14].

In selected cases where chronic pulmonary artery obstruction is evident but the etiology remains uncertain, surgical exploration via PEA can serve both diagnostic and therapeutic purposes. In rare instances, sclerotherapy-related foreign materials may give rise to CTEPD without manifest pulmonary hypertension, yet still present with significant symptoms and anatomical obstruction. In such cases, surgical intervention may be required despite the absence of elevated pulmonary pressures. This reinforces the expanding role of PEA not only as a curative procedure for CTEPH but also as a valuable diagnostic and therapeutic tool in selected cases of symptomatic CTEPD with unclear etiology.

These patients typically presented with isolated, unilateral lobar obstructions, in contrast to the more common bilateral distribution seen in most PEA cases. Intraoperatively, finding a proper dissection plane was often challenging due to the firm adhesions and dense intimal fibrosis induced by prior sclerotherapy. These pathological changes increased the technical difficulty of endarterectomy and heightened the risk of complications, as deviating from the correct dissection plane could jeopardize patient safety. Nevertheless, this subgroup generally consisted of younger individuals with few comorbidities and preserved cardiopulmonary reserve, allowing for favorable postoperative outcomes despite the surgical complexity.

Diagnosing non-thrombotic pulmonary artery obstructions can be challenging, as their clinical presentation often mimics classical pulmonary embolism. Pulmonary symptoms such as dyspnea, chest discomfort, or exercise intolerance are frequently attributed to unrelated causes, particularly in the absence of overt thrombotic risk factors. In such cases, the link between prior venous interventions and pulmonary vascular pathology may be overlooked, underscoring the importance of raising clinician awareness, especially among those performing sclerotherapy.

Given the histopathological confirmation of sclerotherapy-related materials as a potential cause of chronic vascular obstruction, clinicians should maintain a high index of suspicion in similar cases. Therefore, in patients with a history of sclerotherapy who develop unexplained or persistent pulmonary symptoms, the possibility of foreign material-induced vascular obstruction should be considered. Early evaluation in experienced CTEPH centers is recommended to optimize outcomes through timely diagnosis and individualized management.

## Conclusion

This case series highlights a novel and previously undocumented cause of chronic pulmonary artery obstruction: embolization of foreign material following sclerotherapy. Histopathological confirmation of sclerosing agents within the pulmonary vasculature supports the hypothesis that, in rare cases, these substances may lead to persistent vascular obstruction mimicking CTEPH. To the best of our knowledge, this is the first study to report such an association. Recognizing this potential etiology is crucial, particularly in patients with a history of sclerotherapy who present with unexplained pulmonary symptoms or signs of vascular obstruction. A multidisciplinary approach and careful evaluation of patient history are essential to uncover rare etiologies and optimize outcomes.

## Supplementary Information

Supplementary Material 1.

Supplementary Material 2.

## Data Availability

All data generated or analyzed during this study are included in this published article. Additional details are available from the corresponding author on reasonable request.
