# Supplementary material for: Sclerotherapy embolism: a novel etiology for chronic thromboembolic pulmonary disease
Source: BMC Pulm Med. 2025 Dec 10;26:14. doi: 10.1186/s12890-025-04052-7 (PMC12801887; doi:10.1186/s12890-025-04052-7)
Supplement: Supplementary file 1 — Supplementary Material 1. [file 12890_2025_4052_MOESM1_ESM.docx]

Supplementary Table S1. Individual baseline and postoperative data of patients undergoing pulmonary endarterectomy (PEA)

| Patient No | WHO FC (pre → post) | 6MWD (m) pre → post | mPAP (mmHg) pre → post | PVR (dyn·s/cm⁻⁵) pre → post |
| --- | --- | --- | --- | --- |
| 1 | III → I | 350 → 420 | 31 → 17 | 253 → 59 |
| 2 | III → I | 450 → 550 | 20 → 18 | 160 → 145 |
| 3 | III → I | 416 → 500 | 16 → 15 | 114 → 110 |
| 4 | II → I | 310 → 410 | 30 → 15 | 350 → 163 |

*6MWD, six-minute walk distance; mPAP, mean pulmonary artery pressure; PVR, pulmonary vascular resistance; WHO FC, World Health Organization Functional Class.
