# Supplementary material for: Sclerotherapy embolism: a novel etiology for chronic thromboembolic pulmonary disease
Source: BMC Pulm Med. 2025 Dec 10;26:14. doi: 10.1186/s12890-025-04052-7 (PMC12801887; doi:10.1186/s12890-025-04052-7)

**Supplementary Figure S1.** Representative preoperative and postoperative findings of the four patients with chronic thromboembolic pulmonary disease (CTEPD) secondary to sclerotherapy embolism. Each row (Cases 1–4) includes preoperative computed tomography pulmonary angiography (CTPA) demonstrating unilateral lobar obstruction (yellow arrows), the gross appearance of the endarterectomy specimens, and histopathological sections showing granulomatous foreign body reaction with multinucleated giant cells and lymphocytic infiltration. Necrotic material was also observed.


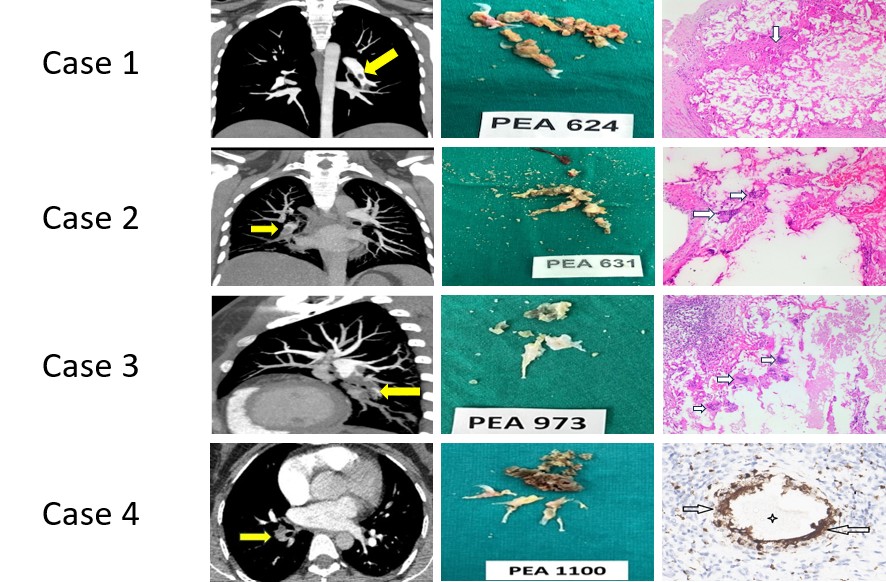

Supplement: Supplementary file 2 — Supplementary Material 2. [file 12890_2025_4052_MOESM2_ESM.docx]
